# Joint Service Chain Orchestration and Computation Offloading via GNN-Based QMIX in Industrial IoT

**DOI:** 10.3390/s26082559

**Published:** 2026-04-21

**Authors:** Xinzhi Huang, Bingxin Tian

**Affiliations:** 1 College of Computer and Network Security, Chengdu University of Technology, Chengdu 610059, China; huangxinzhi@stu.cdut.edu.cn; 2 China Mobile Research Institute, Beijing 100053, China

**Keywords:** industrial internet, edge computing, multi-agent reinforcement learning, graph neural network, task scheduling, resource allocation

## Abstract

In IIoT edge computing, multi-edge server collaborative scheduling faces two core issues due to random task arrivals, heterogeneous resources, and complex topology: traditional model-driven methods cannot make dynamic decisions in dynamic environments, and conventional MARL fails to characterize inter-node topological dependencies and load correlations. To address this, this paper investigates the joint optimization of task offloading, computing resource allocation, and SFC orchestration in IIoT, constructs a cloud-edge-end collaborative architecture, and models the problem as a POMDP to minimize the overall system cost under multiple constraints. A graph-guided value-decomposition MARL method is proposed, which extracts spatial topology and neighborhood-load features of edge nodes via a GNN and combines them with the QMIX framework to realize multi-agent centralized training and distributed execution. Simulations show that the algorithm converges stably under different server scales and task loads, significantly outperforms benchmark algorithms, and can suppress performance degradation in high-load scenarios, demonstrating its robustness and scalability in complex industrial environments.

## 1. Introduction

Industrial Internet of Things (IIoT) has thoroughly reshaped traditional industrial operation modes with its powerful capabilities of real-time data collection, analysis, and decision-making. Furthermore, with the deep integration of artificial intelligence, IIoT has been further extended to key fields such as intelligent manufacturing and smart healthcare, realizing ubiquitous interconnection among devices and dynamic utilization of data [1]. Nevertheless, with the explosive growth of emerging applications such as autonomous driving and industrial automation, the demand for computing resources and low-latency communication in industrial scenarios has surged [2]. However, limited by physical size, battery capacity, and cost, widely distributed Industrial Internet of Things Entities (IIEs) usually face severe bottlenecks in computing and storage resources, making it difficult to independently tackle the challenges posed by these compute-intensive and delay-sensitive tasks [3]. To break this deadlock between resource supply and demand, Mobile Edge Computing (MEC), as an emerging computing paradigm, has emerged. By offloading tasks to edge servers for execution, MEC dynamically alleviates the resource pressure on end devices [4]. However, the overall performance of MEC systems still largely depends on the implementation of dynamic distributed task scheduling and resource allocation strategies to meet stringent Quality of Service (QoS) requirements in heterogeneous and dynamic industrial environments [5,6].

Aiming at the complex resource scheduling and task allocation issues in IIoT, the academic community has conducted extensive explorations, and existing solutions mainly focus on two categories: model-driven mathematical optimization and heuristic search strategies. In dynamic scenarios with energy harvesting devices, Zhang et al. [7] proposed an online dynamic offloading scheme based on Lyapunov optimization theory. By constructing an energy-delay tradeoff model, the scheme minimized the system cost while ensuring the stability of battery queues. In addition, to address the high dynamics of task generation processes and the difficulty in obtaining statistical information in edge computing, Chen et al. [8] applied heuristic algorithms to computation offloading decisions. By decomposing the complex optimization problem into a series of subproblems and solving them with an online distributed strategy, they obtained optimized resource allocation schemes within an acceptable time. These methods have achieved certain effects in static or slowly changing industrial networks, laying a theoretical foundation for early edge computing resource management [9,10].

Although the aforementioned model-driven and heuristic methods have laid a foundation for MEC resource scheduling in IIoT, most studies overlook the decoding-error probability associated with short-blocklength transmission, the delay-violation probability in computation queues in low-latency scenarios, and the two-tier time-allocation trade-off between frames and within a frame [11,12]. Addressing this gap, Wang et al. integrated finite-blocklength information theory with extreme-value theory to characterize the relevant error probabilities, proposed a reliability-optimal offloading framework, and designed low-complexity time-allocation algorithms for both perfect and outdated CSI scenarios, providing crucial support for ultra-reliable MEC offloading [12].

However, when applied to future large-scale, highly dynamic, and hardware-heterogeneous IIoT environments, the aforementioned traditional methods face severe challenges. The first challenge is the contradiction between real-time performance and computational overhead: industrial control is extremely sensitive to latency, while the solution time of precise optimization algorithms grows exponentially as the number of nodes increases, making it difficult to meet millisecond-level requirements for online decision-making [8,13,14,15]. The second challenge is the excessive dependence on accurate environmental models: traditional methods usually assume that channel states and task arrivals follow specific distributions [7], but in real industrial sites, electromagnetic interference and random task flows lead to severe “environmental non-stationarity” and dynamic changes, making models based on static statistical features difficult to adapt to [1,4,16,17]. More importantly, existing schemes often ignore the underlying heterogeneous hardware affinity, simplifying complex computing resources into a single numerical value, and thus fail to capture the differentiated demands of different industrial tasks (e.g., logic control focusing on CPU, visual quality inspection relying on GPU) for specific hardware resources [3,18]. Therefore, there is an urgent need for a real-time decision-making method that can learn autonomously through interaction with the environment without relying on precise mathematical models, making deep reinforcement learning a key technology for solving such problems [19,20,21,22].

In recent years, deep reinforcement learning has been widely introduced into resource orchestration in edge computing due to its powerful perception and decision-making capabilities. The asynchronous reinforcement learning method (A3C) proposed by Mnih et al. [19] effectively solves the policy learning problem in some high-dimensional state spaces through multi-threaded parallel interaction. However, existing Multi-Agent Reinforcement Learning (MARL) schemes still have significant limitations when dealing with complex IIoT collaborative scheduling: on the one hand, most algorithms assume full connectivity or uniform distribution among agents, ignoring the inherent physical spatial topology among edge nodes. The lack of such topological information makes it difficult for agents to perceive the load status of neighboring domains, limiting the effectiveness of collaboration [20,23,24]. On the other hand, in cooperative games aiming to minimize the global total cost (e.g., weighted sum of total latency and energy consumption), existing distributed methods struggle to effectively decompose the global optimization objective, often leading to inconsistencies between local strategies and global goals, thus hindering the convergence of system performance [25,26,27]. In the IIoT scenarios of satellite-assisted mobile edge computing, the dynamic network topology caused by satellite orbital motion and the inaccessibility of global state information due to uneven device distribution in remote areas further exacerbate the decision-making dilemmas of MARL. Although existing studies have achieved global latency optimization under local states by fusing federated learning with deep recurrent Q-learning, partitioning agent clusters by satellite-ground distance, aggregating local models, and capturing temporal features of topological dynamics via recurrent neural networks, the core challenges of multi-satellite collaborative scheduling and non-independent and identically distributed (non-IID) data processing in federated learning remain unsolved [28].

In view of the aforementioned research limitations, this paper clarifies its core positioning in multi-agent collaborative scheduling for IIoT edge computing, clearly distinguishing itself from existing achievements: at the problem level, most existing studies fail to fully consider the coupling of heterogeneous resources, dynamic task flows, and complex topologies in IIoT. Traditional MARL methods lack an effective characterization of inter-node topological dependencies and load correlations and struggle to reasonably decompose the global optimization objective, easily leading to disjointed local decisions and poor global performance. At the algorithm level, existing schemes mostly adopt QMIX alone (only solving objective decomposition) or GNNs alone (only extracting topological features), without deep integration. This paper constructs a cloud-edge-end collaborative optimization model that fits practical industrial scenarios, addressing the insufficient consideration of scenario coupling in existing studies. Meanwhile, it innovatively integrates GNNs’ capability to extract topological and neighborhood-load features into the QMIX architecture, proposing the QGNN algorithm. This approach not only addresses the core pain point of traditional MARL’s lack of global topological perception but also ensures consistency between multi-agent decisions and global objectives, filling the research gap of topology-aware joint optimization of service chain orchestration and computation offloading in high-load, large-scale scenarios. Specifically, the main contributions of this paper are summarized as follows:

(1) To address the challenges brought by heterogeneous resources, limited computing power of edge servers, and dynamic and random requests from factory terminals in the IIoT scenario, this paper proposes a cloud-edge-end collaborative architecture. By deploying virtual network functions (VNFs) on edge servers to reduce network latency, this paper constructs a joint optimization model with the goal of minimizing the total comprehensive cost under the constraints of maximum latency, computing resources, and link transmission bandwidth. The problem is modeled as a Markov decision process (MDP) to jointly optimize task offloading, computing resource allocation, and VNF update decisions.
(2) To address the challenges of complex IIoT network topology and difficulties in distributed multi-agent collaboration, this paper designs a graph-guided value-decomposition multi-agent deep reinforcement learning algorithm. First, the algorithm uses the multi-layer aggregation mechanism of graph neural networks (GNNs) to perceive the spatial topological structure among edge nodes and the neighborhood load information, effectively enhancing the agents’ ability to characterize heterogeneous environments. Second, it adopts the QMIX value decomposition architecture, which accurately decomposes the global optimization objective into local Q-values of each agent through a mixing network satisfying monotonicity constraints, realizing centralized training and distributed execution, thereby significantly reducing collaboration overhead while ensuring global performance.
(3) Simulation results show that the proposed algorithm achieves stable convergence in the dynamically changing IIoT environment. Compared with mainstream benchmark algorithms, it significantly reduces the total comprehensive cost of the system and improves task-processing efficiency.

## 2. System Model and Problem Formulation

**Figure 1:**
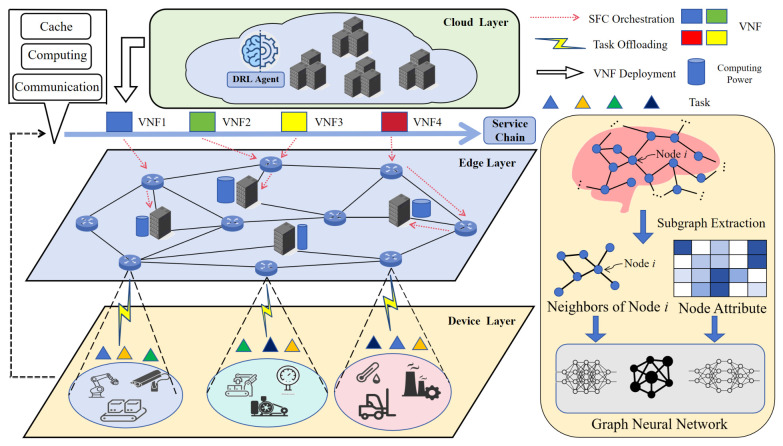
System architecture of cloud-edge-end collaborative computing for IIoT.

This paper considers an enterprise with multiple factories, where each factory area is equipped with a combination of a base station, a gateway, and an edge server. Multiple local Industrial Internet of Things (IIoT) devices in the factory generate computing tasks, which require Virtual Network Functions (VNFs) to be processed. As a core concept in Network Function Virtualization (NFV), VNFs deploy traditional network functions (e.g., firewalls, routers, load balancers) implemented by dedicated hardware in software form on general computing platforms, thereby enabling flexible scheduling and resource sharing of computing and network functions. Table 1 summarizes the key mathematical notations and their definitions, providing a unified reference for subsequent system modeling, algorithm design, and theoretical derivations. Figure 1 presents the cloud-edge-end collaborative computing architecture for industrial IoT, which forms the foundational system scenario for our scheduling and optimization tasks.

Edge servers have limited computing and storage capabilities, and they can download the VNFs required for executing specific tasks from the software library of the cloud center. The factory itself also has a certain level of computing capability. In the scenario considered in this paper, there is a central cloud server with a VNF software library, which can pre-deploy VNFs to edge servers. A total of *K* IIoT devices are distributed in the system, denoted by the set K={1,2,…,K}. A total of *N* combinations of base stations, gateways, and edge servers are distributed across various factory areas, denoted by the set N={1,2,…,N}, with n≤k. It is assumed that there are a total of *F* types of computing tasks that can be generated by the terminals, denoted by the set F={1,2,…,F}. Each task f∈F is defined as $f=\{I_{f},S_{f},D_{f}\}$, where $I_{f}$ represents the size of user data for task type *f*, $S_{f}$ represents the CPU cycles required to execute task *f*, and $D_{f}$ represents the size of the VNF required for task type *f*; each task request corresponds to one VNF. It is assumed that the length of each time slot in the system is τ, and the time sequence is denoted as *t*. At the start of time slot *t*, IIoT device *k* generates $N_{k}^{f}\left(t\right)$ tasks of type *f*. At the end of each time slot, edge servers update their deployment space and actively deploy the corresponding VNFs from the software library of the cloud server. A reasonable caching strategy helps improve task execution efficiency and system performance. Tasks in this paper can be executed entirely locally, offloaded to the edge for execution, and after offloading to the edge, computing resources can be borrowed among multiple edge servers. During computation offloading, if the required VNF exists, the task data is transmitted directly; if not, the VNF must first be downloaded from the cloud server’s software library to the edge server, followed by the transmission of task data and task execution.

### 2.1. Network Model

The system state is modeled as an undirected graph, where each combination of a base station, gateway, and edge server constitutes an edge node. The distances between these three components are short and connected via wired links, so this delay is neglected. At the *t*-th time slot, for task *f*, the system state can be represented by a weighted undirected graph:

(1) $$G_{t}^{f}=\left(V_{t}^{f},E_{t}^{f}\right),$$

where $V_{t}^{f}$ is the vertex set for task *f* at time slot *t*, representing all edge nodes; $E_{t}^{f}$ is the edge set, representing the interconnections between edge nodes for task *f* at time slot *t*.

Each vertex is associated with two attributes: $\left\{N_{n}^{f}\left(t\right),V_{n}^{f}\left(t\right)\right\}$. Here, $N_{n}^{f}\left(t\right)$ denotes the number of type-*f* task requests received by the *n*-th edge server at time slot *t*; $Q_{n}^{f}\left(t\right)$ denotes the computing power allocated by the edge server corresponding to edge node *n* for type-*f* services. Each edge $e_{n,n^{\prime }}^{f}\in E_{t}^{f}$ is associated with two attributes: $\left\{R_{n,n^{\prime }}^{f}\left(t\right),E_{n,n^{\prime }}^{f}\right\}$. Here, $R_{n,n^{\prime }}^{f}\left(t\right)$ denotes the available bandwidth allocated to task *f* between nodes *n* and $n^{\prime }$ at time slot *t*; $E_{n,n^{\prime }}^{f}$ denotes the transmission cost of transmitting one type-*f* task request between the two nodes.

For each node $n\in V_{t}^{f}$, this paper imposes the following constraint:

(2) $$\sum_{f\in F}Q_{n}^{f}\left(t\right)\leq Q_{n}^{\max},\;\forall n\in V_{t}^{f},\forall t\in T$$

where $Q_{n}^{f}\left(t\right)$ is the computing power allocated by node *n* to task *f* at time slot *t*, and $Q_{n}^{\max}$ is the maximum computing power of node *n*.

For each edge $e_{n,n^{\prime }}^{f}\in E_{t}^{f}$, this paper imposes the following bandwidth constraint:

(3) $$\sum_{f\in F}R_{n,n^{\prime }}^{f}\left(t\right)\leq R_{n,n^{\prime }}^{\max},\;\forall \left(n,n^{\prime }\right)\in E_{t}^{f},\forall t\in T$$

where $R_{n,n^{\prime }}^{f}\left(t\right)$ is the bandwidth allocated to task *f* between node *n* and node $n^{\prime }$ at time slot *t*, and $R_{n,n^{\prime }}^{\max}$ is the maximum bandwidth between node *n* and node $n^{\prime }$.

### 2.2. Communication Model

In the considered scenario, the response delay of tasks is affected by scheduling and computing delays.

Assume that the transmission from IIoT device *k* to its corresponding edge gateway is wireless, and its maximum uplink rate can be determined by the Shannon formula. Then, the achievable upload data rate of device *k* at time slot *t* is as follows:

(4) $$r_{k}\left(t\right)=\frac{W}{M(t)}\log\left(1+\frac{P_{k}G_{k}\left(t\right)}{\frac{W}{M}N_{0}}\right),$$

where M(t) is the number of IIoT devices performing offloading operations at time slot *t*; $P_{k}$ is the transmit power of device *k* for uploading data; $G_{k}\left(t\right)$ denotes the wireless channel gain of IIoT device *k* at time slot *t*, whose dynamicity mainly stems from the inherent Rayleigh fading characteristic of wireless channels in industrial scenarios. Real-time fluctuations of channel gain are caused by multipath propagation, slight device mobility, and industrial electromagnetic interference, which is the core physical cause of the dynamicity of IIoT network connectivity. Meanwhile, the task request arrival rate of industrial terminals follows a Poisson process, and the generated quantity and type of tasks change in real time with industrial production processes. The random fluctuation of request traffic further exacerbates the dynamicity of IIoT network connectivity. Together, these two aspects form the practical basis for the high dynamicity of IIoT connectivity in this study. $N_{0}$ is the white noise of the Gaussian channel. The rate at which edge server *n* downloads VNFs from the cloud center is set as $r_{n}\left(t\right)$:

(5) $$r_{n}\left(t\right)=\frac{W}{M(t)}\log\left(1+\frac{P_{n}G_{n}\left(t\right)}{\frac{W}{M}N_{0}}\right),$$

where M(t) is the number of edge servers performing download operations at time slot *t*; $P_{n}$ is the transmit power of edge server *n* for uploading data; and $G_{n}\left(t\right)$ is the channel gain of *n* in the wireless channel.

In this paper, let S be the set of edge server pairs where scheduling occurs. If (m,n)∈S, it indicates that there is scheduling from gateway *m* to server *n*. The corresponding number of scheduled tasks of type *f* is $N_{m,n}^{f}\left(t\right)$. The offloading delay from edge node *m* to edge node *n* at time slot *t* is as follows:

(6) $$T_{m,n}^{f,\mathrm{tran}}\left(t\right)=\frac{N_{m,n}^{f}\left(t\right)I_{f}}{R_{m,n}^{f}\left(t\right)}.$$

Therefore, the total delay of inter-edge transmission at time slot *t* is as follows:

(7) $$T_{f}^{\mathrm{tran}}\left(t\right)=\max\left\{T_{m,n}^{\mathrm{tran}}\left(f,t\right)|\left(m,n\right)\in S\right\}.$$

Furthermore, it is known that the scheduling energy consumption from gateway *m* to edge server *n* at time slot *t* is a constant $E_{m,n}^{f}$. Then, the transmission energy consumption of this link at time slot *t* is as follows:

(8) $$E_{m,n}^{f,\mathrm{tran}}\left(t\right)=N_{m,n}^{f}\left(t\right)E_{m,n}^{f}.$$

Therefore, the total inter-edge transmission energy consumption at time slot *t* is as follows:

(9) $$E_{f}^{\mathrm{tran}}\left(t\right)=\sum_{(m,n)\in S}E_{m,n}^{f,\mathrm{tran}}\left(t\right).$$

### 2.3. VNF Model

At time slot *t*, the caching decision of the VNF required by task *f* on edge server *n* is denoted by $b_{f,n}^{\left(t\right)}\in \left\{0,1\right\}$. $b_{f,n}^{\left(t\right)}=1$ indicates that edge server *n* has deployed the VNF for executing task *f* at time slot *t*; $b_{f,n}^{\left(t\right)}=0$ indicates that the edge server has not deployed the VNF for executing this task type at this time slot. $D_{f}$ denotes the size of the VNF required by task type *f*. The deployment capacity of the edge server is denoted by $C_{n}^{max}$. At any time slot, the size of data deployed on the server should not exceed its capacity $C_{n}^{max}$, i.e.,

(10) $$\sum_{f\in F}b_{f,n}^{\left(t\right)}D_{f}\leq C_{n}^{max},\;\forall t\in T,\forall n\in N.$$

At time slot *t*, the update strategy of server *n* for *F* types of VNFs can be denoted by $B_{n}^{\left(t\right)}=\left\{\beta_{1,n}^{\left(t\right)},\beta_{2,n}^{\left(t\right)},\ldots ,\beta_{F,n}^{\left(t\right)}\right\}$. Let $\beta_{f,n}^{\left(t\right)}\in \left\{-1,0,1\right\}$ represent the VNF deployment update action of task *f* on edge server *n* at time slot *t*. $\beta_{f,n}^{\left(t\right)}=1$ indicates that the VNF for executing task type *f* will be added to the server’s deployment at time slot t+1; $\beta_{f,n}^{\left(t\right)}=0$ indicates that the deployment state of the VNF for executing task type *f* in the server’s deployment remains unchanged at time slot t+1; $\beta_{f,n}^{\left(t\right)}=-1$ indicates that the VNF required by task type *f* will be removed from edge server *n* at time slot t+1. Therefore, the deployment state of task type *f* on edge server *n* at time slot t+1 is as follows:

(11) $$b_{f,n}^{\left(t+1\right)}=b_{f,n}^{\left(t\right)}+\beta_{f,n}^{\left(t\right)},$$

where the edge server cannot remove non-deployed VNFs, so the following should be satisfied:

(12) $$\beta_{f,n}^{\left(t\right)}\geq -b_{f,n}^{\left(t\right)}.$$

At time slot *t*, the terminal requests of server *n* for *F* types of VNFs are denoted by $U_{t}=\left\{\mu_{1}^{\left(t\right)},\mu_{2}^{\left(t\right)},\ldots ,\mu_{K}^{\left(t\right)}\right\}$. Let $\mu_{k}^{\left(t\right)}\in \bar{F}$ ($\bar{F}=\left\{0\right\}\cup F$) represent the computing task request state of terminal *k* at time slot *t*. $\mu_{k}^{\left(t\right)}=0$ indicates that terminal *k* does not generate a computing task request at time slot *t*, and $\mu_{k}^{\left(t\right)}=f\in F$ indicates that terminal *k* generates a request to execute task type *f* at time slot *t*. Furthermore, it is assumed that the task request state of the terminal at time slot t+1 is only affected by its task request state at time slot *t*. We first define

(13) $$p_{k}\left(i,j\right)≜\mathrm{Pr}\left(\mu_{k}^{\left(t+1\right)}=j|\mu_{k}^{\left(t\right)}=i\right)$$

Then, the rules for the transition probability of the task request $\mu_{k}^{\left(t\right)}$ of terminal *k* at time slot *t* to the task request $\mu_{k}^{\left(t+1\right)}$ at time slot t+1 are as follows:

(14) $${\left(P_{k}\right)}_{i,j}=\left\{\begin{array}{cc} R, & \mathrm{if}\;i\in F,j=0\\ \left(1-R\right)\frac{j^{-\delta }}{\sum_{j^{\prime }\in F}j^{\prime -\delta }}, & \mathrm{if}\;i=0,j\in F\\ 1-R, & \mathrm{otherwise}. \end{array}\right.$$

where each task is associated with *N* artificially set neighboring tasks. *R* represents the probability that there is any task request in the current time slot, but no request in the next time slot; when i=0,j∈F, the state transition probability follows a Zipf distribution, which is determined by the parameter δ.

At the beginning of each time slot, each IIoT device generates a computing task, which belongs to a certain task category in the task category library F. This chapter assumes that, regardless of the task execution method adopted, each computing task must be executed before the end of the corresponding time slot. To meet this requirement, the length of each time slot (i.e., the value of τ) can be adjusted to ensure that tasks can be completed within the specified time limit.

### 2.4. Computing Model

In this paper, $\alpha_{k}^{\left(t\right)}\in \left\{0,1\right\}$ is used to represent the offloading mode of IIoT device *k* at time slot *t*, and the offloading decision is defined as $\lambda =\{\alpha_{1}^{\left(t\right)},\alpha_{2}^{\left(t\right)},\ldots ,\alpha_{K}^{\left(t\right)}\}$, where $\alpha_{k}^{\left(t\right)}=0$ indicates that device *k* executes tasks through its own resources; $\alpha_{k}^{\left(t\right)}=1$ indicates that device *k* offloads tasks to the server for execution.

This paper neglects the delay and energy consumption generated during the process of terminals sending task requests to servers and servers returning whether the corresponding VNF has been deployed.

#### 2.4.1. Local Computing Mode

If task *f* is selected to be computed only locally, the local execution latency is as follows:

(15) $$T_{k,f}^{\mathrm{local}}\left(t\right)=\left(1-\alpha_{k}\left(t\right)\right)\cdot \frac{N_{k}^{f}\left(t\right)S_{f}}{Q_{k}^{L}},$$

where $Q_{k}^{L}$ is the local computing capability of IIoT device *k*. The energy consumption required for task *f* execution is expressed as follows:

(16) $$E_{k,f}^{\mathrm{Local}}\left(t\right)=\left(1-\alpha_{k}\left(t\right)\right)\cdot N_{k}^{f}\left(t\right)Z_{k}S_{f},$$

where $Z_{k}$ is the energy consumption required per CPU cycle. According to the literature, this chapter sets $Z_{k}=10^{-27}{\left(Q_{k}^{L}\right)}^{2}$.

#### 2.4.2. Edge Computing Mode (Offloading Computing Mode)

If IIoT device *k* executes task *f* by offloading it to an edge server, the entire offloading process is as follows:

First, *k* uploads data to the base station via wireless transmission. The maximum uplink rate from IIoT device *k* to the edge gateway is $r_{k}\left(t\right)$, so the transmission latency from industrial device *k* to the corresponding edge gateway is as follows:

(17) $$T_{k,f}^{\mathrm{tran}}\left(t\right)=\alpha_{k}\left(t\right)\cdot \frac{N_{k}^{f}\left(t\right)I_{f}}{r_{k}\left(t\right)}.$$

Then, the base station transmits the data to the edge server via wired transmission. The edge server adjusts computing resources according to the offloading strategy to execute the task, and finally returns the result to the corresponding device. When offloading is selected, IIoT device *k* uploads $N_{k}^{f}\left(t\right)$ tasks *f* to the corresponding edge server *n* at time slot *t*, which means the number of tasks *f* on server *n* at time slot *t* increases by $N_{k}^{f}\left(t\right)$. Then, scheduling between nodes can be performed. As defined earlier, the number of tasks *f* scheduled from node *m* to node *n* at time slot *t* is $N_{m,n}^{f}\left(t\right)$. Then the number at this time slot is as follows:

(18) $$N_{n}^{f}\left(t\right)=N_{k}^{f}\left(t\right)-\sum_{(m,n)\in S}N_{m,n}^{f}\left(t\right),$$

where $Q_{n}^{f}\left(t\right)$ is the computing resource allocated by edge server *n* for executing the offloaded computing task *f* generated by device *n* (unit: CPU cycles/s). The following constraint must be satisfied:

(19) $$\sum_{f\in F}Q_{n}^{f}\left(t\right)\leq Q_{n},\;\forall n\in V_{t}^{f},$$

i.e., the total computing resources of the edge server allocated to offloaded tasks generated by all devices do not exceed $Q_{n}$. Then the task processing latency of server *n* for task *f* at time slot *t* can be expressed as follows:

(20) $$T_{n,f}^{\mathrm{hand}}\left(t\right)=\alpha_{k}\left(t\right)\cdot \frac{N_{n}^{f}\left(t\right)S_{f}}{Q_{n}^{f}\left(t\right)}.$$

The total computing resources of the edge server that are allocated to offloaded tasks generated by all devices do not exceed *Q*.

(21) $$E_{n,f}^{\mathrm{hand}}\left(t\right)=\alpha_{k}\left(t\right)\cdot N_{n}^{f}\left(t\right)S_{f}Z_{k},$$

where $Z_{n}$ is the energy consumption required per CPU cycle, and this chapter sets $Z_{n}=10^{-27}{\left(Q_{n}^{f}\right)}^{2}$.

Since computing on the edge server requires a VNF, if the computation is performed when the VNF is not deployed, the latency for edge server *n* to download the VNF corresponding to task *f* from the cloud center is as follows:

(22) $$T_{n,f}^{\mathrm{down}}\left(t\right)=\alpha_{k}\left(t\right)\cdot \left(1-b_{f,n}^{\left(t\right)}\right)\cdot \frac{N_{n}^{f}\left(t\right)D_{f}}{r_{n}\left(t\right)}.$$

Then this latency can be expressed as follows:

(23) $$T_{n,f}^{\mathrm{off}}\left(t\right)=T_{n,f}^{\mathrm{hand}}\left(t\right)+T_{n,f}^{\mathrm{down}}\left(t\right).$$

When computing offloading, the power used by edge server *n* for downloading data is $P_{n}\left(t\right)$, and the corresponding energy consumption is as follows:

(24) $$E_{n,f}^{\mathrm{down}}\left(t\right)=P_{n}\left(t\right)\cdot T_{n,f}^{\mathrm{down}}\left(t\right).$$

Then the energy consumption of edge server *n* can be expressed as follows:

(25) $$E_{n,f}^{\mathrm{off}}\left(t\right)=E_{n,f}^{\mathrm{hand}}\left(t\right)+E_{n,f}^{\mathrm{down}}\left(t\right).$$

In addition, when computing offloading, the power used for uploading data is $P_{k}\left(t\right)$, and the corresponding energy consumption is as follows:

(26) $$E_{k,f}^{\mathrm{tran}}\left(t\right)=\alpha_{k}\left(t\right)\cdot P_{k}\left(t\right)\cdot \frac{N_{n}^{f}\left(t\right)I_{f}}{r_{k}\left(t\right)}.$$

For ease of calculation, we express the latency related to device *k* as follows:

(27) $$T_{k,f}^{L}\left(t\right)=T_{k,f}^{\mathrm{local}}\left(t\right)+T_{k,f}^{\mathrm{tran}}\left(t\right).$$

The energy consumption related to device *k* is expressed as follows:

(28) $$E_{k,f}^{L}\left(t\right)=E_{k,f}^{\mathrm{local}}\left(t\right)+E_{k,f}^{\mathrm{tran}}\left(t\right).$$

### 2.5. Total Cost and Optimization Problem

The total cost is composed of the weighted sum of total latency and total energy consumption. The total latency is defined as follows:

(29) $$T\left(t\right)=\sum_{f\in F}\left(T_{f}^{\mathrm{tran}}\left(t\right)+\sum_{k\in K}T_{k,f}^{L}\left(t\right)+\sum_{n\in N}T_{n,f}^{\mathrm{off}}\left(t\right)\right),$$

and the total energy consumption is defined as follows:

(30) $$E\left(t\right)=\sum_{f\in F}\left(E_{f}^{\mathrm{tran}}\left(t\right)+\sum_{k\in K}E_{k,f}^{L}\left(t\right)+\sum_{n\in N}E_{n,f}^{\mathrm{off}}\left(t\right)\right).$$

This paper focuses on the joint optimization of latency and energy consumption with equal weights, without considering the unit of total cost. Latency and energy consumption are calculated independently, and experimental results show that the orders of magnitude of these two physical quantities are close. Therefore, this paper controls their numerical proportion through an appropriate weight to avoid an excessively large value of either quantity. We define the weight as γ, and the total weighted cost is given by:

(31) $$A\left(t\right)=\omega_{t}T\left(t\right)+\left(1-\omega_{e}\right)E\left(t\right).$$

The goal of this paper is to minimize the total cost over all time slots. Thus, the optimization problem *P* can be formulated as follows:

(32) $$P:\min_{\alpha_{k}^{\left(t\right)},\beta_{f,n}^{\left(t\right)},N_{n,n^{\prime }}^{f}\left(t\right)}\sum_{t=1}^{T}A\left(t\right),$$

subject to the following constraints:

(33) $$\begin{array}{cc} \; & C_{1}:\sum_{f\in F}V_{n}^{f}\left(t\right)\leq V_{n}^{\max},\;\forall n\in V_{t}^{f},\forall t\in T, \end{array}$$

(34) $$\begin{array}{cc} \; & C_{2}:\sum_{f\in F}R_{n,n^{\prime }}^{f}\left(t\right)\leq R_{n,n^{\prime }}^{\max},\;\forall \left(n,n^{\prime }\right)\in E_{t}^{f},\forall t\in T, \end{array}$$

(35) $$\begin{array}{cc} \; & C_{3}:\sum_{f\in F}b_{f,n}^{\left(t\right)}D_{f}\leq C_{n}^{\max},\;\forall t\in T,\forall n\in N, \end{array}$$

(36) $$\begin{array}{cc} \; & C_{4}:\beta_{f,n}^{\left(t\right)}\geq -b_{f,n}^{\left(t\right)},\;\forall t\in T,\forall n\in N,\forall f\in F, \end{array}$$

(37) $$\begin{array}{cc} \; & C_{5}:\alpha_{k}^{\left(t\right)}\in \left\{0,1\right\},\;\forall t\in T,\forall k\in K, \end{array}$$

(38) $$\begin{array}{cc} \; & C_{6}:T_{k,f}^{\mathrm{local}}\left(t\right)\leq \tau ,\;\forall t\in T,\forall k\in K,\forall f\in F, \end{array}$$

(39) $$\begin{array}{cc} \; & C_{7}:T_{f}^{\mathrm{tran}}\left(t\right)+T_{n,f}^{\mathrm{off}}\left(t\right)\leq \tau ,\;\forall t\in T,\forall n\in N,\forall f\in F, \end{array}$$

(40) $$\begin{array}{cc} \; & C_{8}:N_{n}^{f}\left(t\right)\geq 0,\;\forall n\in N,\forall f\in F, \end{array}$$

(41) $$\begin{array}{cc} \; & C_{9}:N_{n,n^{\prime }}^{f}\left(t\right)\in \left\{0,1,2,\ldots ,N_{n}^{f}\left(t\right)\right\},\;\forall \left(n,n^{\prime }\right)\in E_{t}^{f},\forall t\in T. \end{array}$$

where $C_{1}$ indicates that, for each node $n\in V_{t}^{f}$, the computing power allocated to task *f* shall not exceed the maximum computing power $V_{n}^{\max}$ of the node. $C_{2}$ indicates that for each edge $e_{n,n^{\prime }}^{f}\in E_{t}^{f}$, the bandwidth allocated to that edge shall not exceed the maximum bandwidth $R_{n,n^{\prime }}^{\max}$ between the two nodes. $C_{3}$ indicates that, at any time slot, the size of the data deployed on the server shall not exceed its storage capacity $C_{n}^{\max}$. $C_{4}$ indicates that non-deployed VNFs cannot be removed. $C_{5}$ ensures that the allocated computing resources do not exceed the maximum computing capacity of the target node. $C_{6}$ indicates that the latency of tasks executed through local computing shall not exceed the time-slot length τ. $C_{7}$ indicates that the sum of all latencies (including data transmission, computation, and caching latency) for tasks executed through offloaded computing shall not exceed the time-slot length τ. $C_{8}$ indicates that the number of tasks at the node after scheduling shall not be less than 0. $C_{9}$ indicates that the optimization variable $N_{n,n^{\prime }}^{f}\left(t\right)$ is a non-negative integer and is less than the number of tasks owned by the node.

## 3. Algorithm Design

The task scheduling and resource allocation problem studied in this paper involves multi-dimensional decision variables such as caching, computing, and scheduling, and the system state changes dynamically over time. The overall optimization problem exhibits significant nonlinear coupling and high-dimensional combinatorial characteristics, and is proven to be a typical NP-hard problem.

Traditional model-driven optimization methods (e.g., linear programming, nonlinear programming, or convex optimization) usually rely on accurate modeling of the system state, task generation mode, and network link conditions. However, in actual industrial IoT scenarios, environmental noise, link congestion, and the randomness of task arrivals lead to high uncertainty in the system model. It is difficult to establish a stable and solvable analytical model, and the computational complexity of solving the problem increases exponentially with the increasing scale of IIoT devices and edge servers, making it impossible to meet the real-time requirements for industrial on-site decision-making.

We propose a graph neural network-enhanced QMIX (QGNN) algorithm by integrating graph neural networks into the QMIX framework for joint task offloading, caching updates, and resource allocation. In this framework, IIoT devices and edge servers are modeled as intelligent agents. By introducing graph neural networks to extract the structural information of the edge network and the neighborhood load information, the agents can perceive global information within their local domain in a distributed manner. Combined with the QMIX value decomposition architecture, the global optimization objective is decomposed into the local optimization objectives of each agent, realizing centralized training and distributed execution.

This method not only overcomes the limitation that traditional reinforcement learning algorithms cannot effectively utilize the structural information of the industrial IoT system, but also solves the problem of inconsistent optimization objectives between local agents and the global system in multi-agent collaboration. It can dynamically learn an adaptive scheduling and resource allocation strategy in the dynamic industrial IoT environment, and finally realize the joint optimization of system delay and energy consumption.

### 3.1. Partially Observable Markov Decision Process Model

There are two types of heterogeneous agents in this paper: one is the IIoT device agent, and the other is the edge server agent. The partially observable Markov decision process in this section can be represented as a triple, including the state space S, action space A, and reward function R.

**Figure 2:**
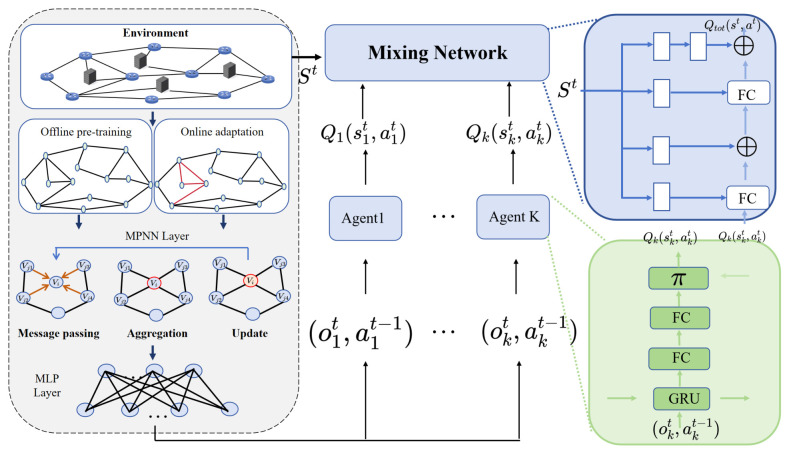
Framework of the graph neural network-enhanced QMIX.

The proposed graph neural network-enhanced QMIX framework is illustrated in Figure 2. It perceives edge-network topology via message passing and enables distributed decision-making for resource allocation and VNF deployment in dynamic industrial scenarios. State: The state of device *k* agent is expressed as follows:

(42) $$S_{k}^{t}=\left\{\mu_{k}^{\left(t\right)},N_{k}^{f}\left(t\right),G_{k}^{\left(t\right)}\right\}$$

where $\mu_{k}\left(t\right)$ is the task type requested by the device, $N_{k}^{f}\left(t\right)$ is the number of tasks for the requested task *f*, and $G_{k}\left(t\right)$ is the channel gain of *k* in the wireless channel.

The state of edge-server agent *n* is expressed as follows:

(43) $$S_{n}^{t}=\left\{N_{n}\left(t\right),Q_{n}^{r}\left(t\right),b_{n}\left(t\right)\right\},$$

where the set $N_{n}\left(t\right)=\left\{N_{n}^{1}\left(t\right),N_{n}^{2}\left(t\right),\ldots ,N_{n}^{F}\left(t\right)\right\}$, and $N_{n}^{f}\left(t\right)$ represents the number of type-*f* tasks received in this time slot. The set $b_{n}\left(t\right)=\left\{b_{1,n}\left(t\right),b_{2,n}\left(t\right),\ldots ,b_{F,n}\left(t\right)\right\}$, and $b_{n}^{f}\left(t\right)$ represents the VNF caching state corresponding to task *f* at this time slot. $Q_{n}^{r}\left(t\right)$ is the remaining computing power of server *n* at this time slot, calculated as follows:

(44) $$Q_{n}^{r}\left(t\right)=Q_{n}\left(t\right)-\sum_{f\in F}Q_{f,n}\left(t\right).$$

This paper defines the global state as follows:

(45) $$S=\left\{S_{k}^{t},S_{n}^{t}|k=1,2,\ldots ,K;n=1,2,\ldots ,N\right\}.$$

Action: The action space of device *k* agent is as follows:

(46) $$a_{k}^{t}=\left\{\alpha_{k}\left(t\right)\right\},$$

where $\alpha_{k}\left(t\right)$ is the offloading decision.

The action space of edge server *n* is as follows:

(47) $$a_{n}^{t}=\left\{Q_{n}\left(t\right),N_{n,n^{\prime }}\left(t\right),\beta_{n}\left(t\right)\right\},$$

where the set $Q_{n}\left(t\right)=\left\{Q_{n}^{1}\left(t\right),Q_{n}^{2}\left(t\right),\ldots ,Q_{n}^{F}\left(t\right)\right\}$, representing the computing resources allocated by edge server *n* for computing task *f*. The set $N_{n,n^{\prime }}\left(t\right)=\left\{N_{n,n^{\prime }}^{1}\left(t\right),N_{n,n^{\prime }}^{2}\left(t\right),\ldots ,N_{n,n^{\prime }}^{F}\left(t\right)\right\}$, representing that server *n* offloads $N_{n,n^{\prime }}^{f}\left(t\right)$ tasks *f* to server $n^{\prime }$. The set $\beta_{n}\left(t\right)=\left\{\beta_{1,n}\left(t\right),\beta_{2,n}\left(t\right),\ldots ,\beta_{F,n}\left(t\right)\right\}$, representing the set of caching actions of edge server *n* for task *f*.

Reward: This paper has a global reward function:

(48) $$\begin{array}{cc} R(t)= & -A\left(t\right)-\lambda_{c}\cdot \sum_{n\in N}\max\left(0,\sum_{f\in F}Q_{f}^{t}\left(t\right)-Q_{n}^{\max}\right)\\ & -\lambda_{s}\cdot \sum_{n\in N}\max\left(0,\sum_{f\in F}b_{f,n}^{\left(t\right)}D_{f}-C_{n}^{\max}\right)\\ & -\lambda_{d}\cdot \sum_{k\in K}\sum_{f\in F}\max\left(0,T_{k,f}^{\left(t\right)}-\tau \right), \end{array}$$

where A(t) is the cost at this time slot, $\lambda_{c}$ is the penalty coefficient for the computing-power constraint, $\lambda_{s}$ is the penalty coefficient for the deployment-capacity constraint, and $\lambda_{d}$ is the penalty coefficient for task completion not within the time slot. And $T_{k,f}^{\left(t\right)}$ is defined as follows:

(49) $$T_{k,f}^{\left(t\right)}=T_{k,f}^{\mathrm{local}}\left(t\right)+T_{k,f}^{\mathrm{tran}}\left(t\right)+T_{n,f}^{\mathrm{off}}\left(t\right).$$

### 3.2. Graph Neural Network Design

Based on this, we construct an undirected graph G=(V,E), where the vertex set V contains all edge nodes, each corresponding to an agent. If there is an available scheduling and communication link between two edge nodes, an edge (m,n) is connected in the graph; otherwise, no direct edge is connected. In this way, the topological structure of the graph reflects the physical connectivity and task transferability between edge nodes.

As shown in Table 2, the GNN feature extraction module adopted in this paper consists of a 5-layer structure, which can efficiently perceive the topological dependencies and node-load information of the edge network. Specifically, the Input Layer takes the original node state vector $x_{t}$ as input; the Message Generation Layer generates intermediate messages by fusing the neighboring node feature $x_{n^{\prime }}^{\left(l-1\right)}$ and edge feature $e_{n^{\prime },n}$ via the ReLU activation function; the Message Aggregation Layer summarizes neighborhood messages through summation to form a unified neighborhood representation; the State Update Layer updates the current node state by combining the aggregated information and the node state from the previous layer $x_{n}^{\left(l-1\right)}$ via the ReLU activation function; the Final Layer outputs the high-dimensional node embedding vector $x_{n}^{\left(L\right)}$, which serves as the input to the subsequent GRU temporal feature extraction module. This structure can effectively encode the key topological information of the industrial edge network, providing structured feature support for multi-agent decision-making.

#### 3.2.1. Node and Edge Feature Construction

The initial feature vector of each graph node *n* is $x_{n}^{t}$, which describes the current resource and load status of the edge node. This includes the remaining computing power ratio:

(50) $${\tilde{Q}}_{n}\left(t\right)=\frac{Q_{n}-Q_{n}^{r}\left(t\right)}{Q_{n}},$$

and the Virtual Network Function (VNF) caching status:

(51) $$b_{n}\left(t\right)={\left[b_{n,1}\left(t\right),b_{n,2}\left(t\right),\ldots ,b_{n,F}\left(t\right)\right]}^{⊤},$$

where $b_{n,f}\left(t\right)\in \left\{0,1\right\}$.

The number of tasks of each type currently received by node *n* is as follows:

(52) $$n_{n}\left(t\right)=\left[N_{n}^{1}\left(t\right),N_{n}^{2}\left(t\right),\ldots ,N_{n}^{F}\left(t\right)\right]$$

These are comprehensively combined to form the node feature:

(53) $$x_{n}^{t}=\mathrm{concat}\left({\tilde{Q}}_{n}\left(t\right),b_{n}\left(t\right),n_{n}\left(t\right)\right)$$

The features of each edge in the graph include the fixed total bandwidth and energy-consumption values. For edge $\left(n,n^{\prime }\right)$, there is a fixed maximum bandwidth of $R_{n,n^{\prime }}^{\max}$. $E_{n,n^{\prime }}^{f}$ represents the transmission cost for a type-*f* task request between two nodes, so we have the following:

(54) $$E_{n,n^{\prime }}=\left[E_{n,n^{\prime }}^{f}|f=1,2,\ldots ,F\right]$$

Therefore, the edge feature vector can be constructed as a vector containing bandwidth and transmission cost:

(55) $$e_{n,n^{\prime }}^{t}=\mathrm{concat}\left(R_{n,n^{\prime }}^{\max},E_{n,n^{\prime }}\right)$$

#### 3.2.2. Aggregation Update

The aggregation operation is performed in each layer of the GNN, and the process can be decomposed into three consecutive, differentiable steps: message generation, message aggregation, and state update.

Since the input layer of the GNN is node features, the initial node embedding is defined as follows:

(56) $$x_{n}^{\left(0\right)}=x_{n}^{t}$$

For each edge server node *n* in the graph and any of its neighbor nodes $n^{\prime }$, we generate a message sent from $n^{\prime }$ to *n*. This message is determined by a message passing function:

(57) $$m_{n^{\prime },n}^{\left(l\right)}=\mathrm{ReLU}\left(W_{\mathrm{msg}}^{\left(l\right)}\left[x_{n^{\prime }}^{\left(l-1\right)}Pe_{n^{\prime },n}^{t}\right]+b_{\mathrm{msg}}^{\left(l\right)}\right)$$

where $x_{n^{\prime }}^{\left(l-1\right)}$ is the embedding vector of neighbor node $n^{\prime }$ at layer l−1; $e_{n^{\prime },n}^{t}$ is the feature vector of edge $\left(n^{\prime },n\right)$; P denotes the vector concatenation operation; $W_{\mathrm{msg}}^{\left(l\right)}$ and $b_{\mathrm{msg}}^{\left(l\right)}$ are the learnable weight matrix and bias term at layer *l*; ReLU(·) is the activation function.

Node *n* aggregates all messages passed from its neighbors to obtain the aggregated information $x_{n}^{\left(l\right)}$. This paper adopts a sum aggregation method:

(58) $$x_{n}^{\left(l\right)}=\sum_{n^{\prime }\in N\left(n\right)}m_{n^{\prime },n}^{\left(l\right)}$$

Node *n* uses the aggregated information $x_{n}^{\left(l\right)}$ and its own previous layer state $x_{n}^{\left(l-1\right)}$ to calculate its new embedding vector $x_{n}^{\left(l\right)}$ at the current GNN layer through a learnable update function:

(59) $$x_{n}^{\left(l\right)}=\mathrm{ReLU}\left(W_{\mathrm{upd}}^{\left(l\right)}\left[x_{n}^{\left(l-1\right)}\|x_{n}^{\left(l\right)}\right]+b_{\mathrm{upd}}^{\left(l\right)}\right)$$

where $W_{\mathrm{upd}}^{\left(l\right)}$ and $b_{\mathrm{upd}}^{\left(l\right)}$ are the parameters of the update function.

After *L* layers of GNN aggregation, each edge server agent *n* will obtain a final embedding vector that integrates network topology and neighborhood state information:

(60) $$z_{n}=x_{n}^{\left(L\right)}$$

#### 3.2.3. Temporal Feature Memory and Q-Value Generation

Considering the partial observability of the industrial IoT environment, relying solely on the current spatial-topology features is insufficient to capture the temporal patterns of task arrivals. Therefore, this paper introduces a gated recurrent unit (GRU) at the output of the GNN. The spatial embedding vector $h_{n}^{\left(L\right)}\left(t\right)$ obtained by GNN aggregation is input to the GRU to update the hidden state of the agent:

(61) $$h_{n}^{\mathrm{hidden}}\left(t\right)=\mathrm{GRU}\left(h_{n}^{\left(L\right)}\left(t\right),h_{n}^{\mathrm{hidden}}\left(t-1\right)\right)$$

Finally, the hidden state is input into a fully connected layer to generate the local Q-values of the current time slot for subsequent use by the mixing network:

(62) $$Q_{n}\left(\tau_{n},\cdot \right)=\mathrm{MLP}\left(h_{n}^{\mathrm{hidden}}\left(t\right)\right)$$

### 3.3. QMIX Design

This paper adopts the QMIX algorithm to realize multi-agent value decomposition. Each agent *i* has a local Q-value function $Q_{i}\left(\tau^{i},a^{i}\right)$, which evaluates the long-term expected return of action $a^{i}$ based on the action-observation history $\tau^{i}$ of the agent.

To measure the quality of joint actions during the centralized training phase, a centralized mixing network is used to decompose the global Q-value $Q_{\mathrm{tot}}\left(\tau ,u,s\right)$ of joint actions into a non-linear combination of local Q-values of each agent:

(63) $$Q_{\mathrm{tot}}\left(\tau ,u,s\right)=\mathrm{Mixer}\left(Q_{1}\left(\tau^{1},u^{1}\right),\ldots ,Q_{N}\left(\tau^{N},u^{N}\right);s\right)$$

where *s* is the global state (only available during training). The structure of the mixing network is constrained to satisfy monotonicity:

(64) $$\frac{\partial Q_{\mathrm{tot}}}{\partial Q_{i}}\geq 0,\;\forall i\in N\cup K$$

This constraint ensures that agents can necessarily maximize the global Q-value by greedily maximizing their local Q-values.

The system adopts a centralized training paradigm, a core training logic of the QMIX framework that integrates local Q-values of all agents via a centralized mixing network during training, while introducing globally accessible state information (only available in the training phase) to constrain the value decomposition process, ensuring consistency between local decisions and the global optimization objective, with the goal of minimizing the temporal difference (TD) error. The loss function is defined as the mean squared error between the forward global Q-value $Q_{\mathrm{tot}}$ and the target global Q-value $y^{\mathrm{tot}}$:

(65) $$L\left(\theta \right)=\sum_{b=1}^{B}{\left(y_{b}^{\mathrm{tot}}-Q_{\mathrm{tot}}\left(\tau ,u,s;\theta \right)\right)}^{2}$$

where *B* is the batch size, and θ contains all trainable parameters of the GNN, GRU, and mixing network. The calculation formula for the target value $y^{\mathrm{tot}}$ is as follows:

(66) $$y^{\mathrm{tot}}=r\left(t\right)+\gamma \max_{u^{\prime }}Q_{\mathrm{tot}}\left(\tau^{\prime },u^{\prime },s^{\prime };\theta^{-}\right)$$

where r(t) is the global immediate reward fed back by the environment; γ is the discount factor; $s^{\prime }$ is the state at the next time step; $\theta^{-}$ is the parameter of the target network, which is periodically copied from the online network to stabilize the training process.

After training is completed, the mixing network is removed. Each edge server agent operates independently and selects the action that maximizes its local Q-value for execution based only on its local observation history $\tau_{n}$, i.e.,

(67) $$a_{n}^{*}=\arg\max_{a^{\prime }}Q_{n}\left(\tau_{n},a^{\prime }\right)$$

The computational complexity of the QMIX algorithm adopted in this paper is mainly reflected in the two phases of centralized training and distributed execution, with the total number of agents $N_{agent}$ (including IIoT device agents and edge server agents), the network hidden layer dimension $d_{h}$, and the total number of training episodes $T_{epis}$ as the core parameters. For the centralized training phase, the time complexity is $\begin{array}{c} O({\left(N_{agent}\cdot d_{h}\right)}^{2}\cdot T_{epis}) \end{array}$ due to the joint optimization of the local Q-networks of all agents and the global mixing network, where $\begin{array}{c} {\left(N_{agent}\cdot d_{h}\right)}^{2} \end{array}$ corresponds to the parameter update overhead of forward and backward propagation of the neural network, and $T_{epis}$ is the cumulative overhead of training iterations. In the distributed execution phase, the global mixing network is removed, and each agent makes decisions only based on its local Q-network, with the time complexity of a single agent being $\begin{array}{c} O(d_{h}^{2}) \end{array}$. It is worth noting that the monotonicity constraint of the mixing network is only realized through the weight matrix design without introducing additional computational complexity, and the overall algorithm complexity increases polynomially with the number of agents, which is suitable for the actual deployment scenario of edge computing in the Industrial Internet of Things.

## 4. Simulation Tests

In this section, the performance of the proposed algorithm (abbreviated as QGNN in the simulation figures) is systematically evaluated through simulation experiments.

To comprehensively verify the effectiveness and stability of the algorithm, the experiments are mainly conducted from the following three aspects:

1. **Algorithm convergence performance analysis:** Investigate the training stability and convergence speed of QGNN under different server scales and hyperparameter configurations.
2. **Comparative algorithm performance evaluation:** Compare QGNN with GCN (graph convolutional network) [29], RNN (recurrent neural network) [30], and random strategies to verify its advantages in complex scenarios.
3. **System performance indicator analysis:** Compare key indicators such as system overhead, average latency, and average energy consumption under different network scales and task load conditions.

The simulation environment parameters and reinforcement learning-related hyperparameters are summarized in Table 3.

The industrial edge environment parameters (including edge-server computing power, local-device computing power, task data size, etc.) are all based on the industrial scenario configuration adopted by Li et al. in their research on shared VNF deployment in mobile edge computing [31]. Focusing on practical IIoT multi-server collaborative scheduling scenarios, the parameter settings in this study fully align with the resource constraints and task characteristics of edge nodes in industrial environments (such as differences in device computing power and the dynamic range of task data sizes), ensuring the realism and engineering applicability of the experimental environment parameters and providing scenario-level support for the validity of the experimental results.

The reinforcement learning hyperparameters (including learning rate, discount factor, experience replay buffer size, etc.) are all drawn from the mature settings of the distributed DRL framework proposed by Wang et al. for IoT application scheduling in edge-cloud environments [32]. Specifically designed for dynamic and heterogeneous edge computing scenarios, this framework’s hyperparameters have been fully validated to effectively balance the convergence speed and stability of model training. They ensure that the multi-agent reinforcement learning model achieves efficient decision-making under dynamic task flows and resource fluctuations in industrial scenarios, while improving the reproducibility and cross-scenario comparison value of this experiment.

**Figure 3:**
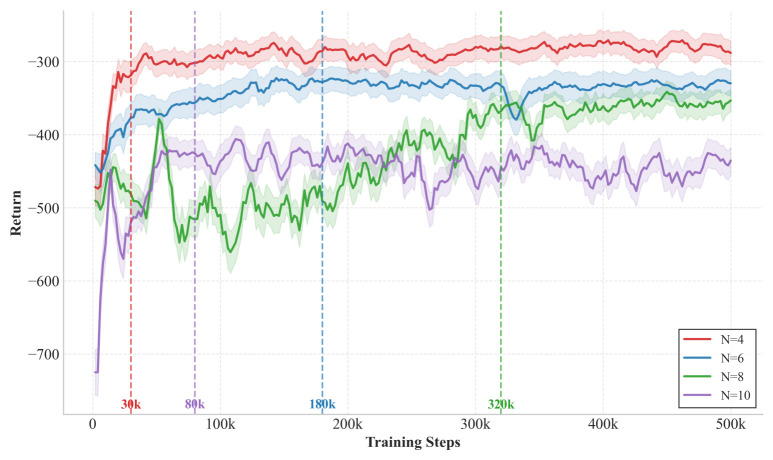
Average reward convergence of QGNN under different edge server scales (N = 4, 6, 8, 10).

Figure 3 shows the average reward convergence curve of the QGNN algorithm under different numbers of servers (N = 4, 6, 8, 10). It can be seen that with the increase in training steps, the rewards under all scales continue to rise and tend to stabilize after about steps, indicating that the algorithm can learn stable and effective scheduling strategies. As the number of servers N increases, the final convergent reward value decreases overall. This is because the system reward is defined as the negative value of the total overhead, and the expansion of scale brings about the cumulative effect of tasks and energy consumption. Nevertheless, all curves can converge smoothly under different scales, verifying the stability and scalability of QGNN in large-scale scenarios.

We further statistically analyze the convergence steps of QGNN under different server scales. Although QGNN integrates GNN, GRU, and QMIX mechanisms with certain model complexity, it can converge within reasonable steps (30 k to 320 k steps) across all scales, without exponential growth of training temporal overhead, verifying its scalability and training efficiency in complex systems.

**Figure 4:**
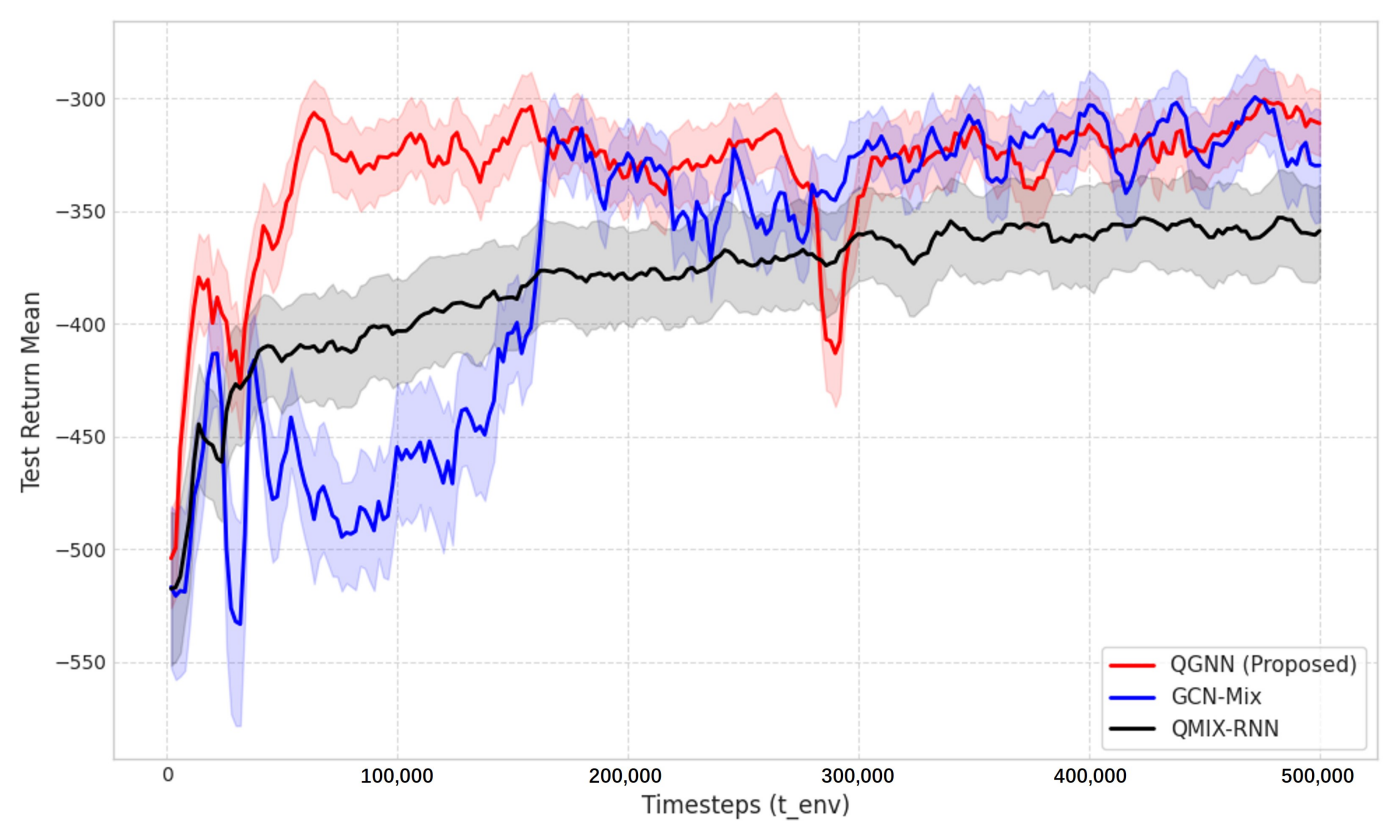
Convergence performance comparison between QGNN and benchmark algorithms (GCN, RNN) with N = 6.

Figure 4 shows the average reward convergence curves of the proposed QGNN and comparison algorithms (GCN and RNN) when the number of edge servers *N* = 6. It can be seen that the reward values of the three algorithms gradually increase with the increase in training steps and tend to stabilize after about steps, indicating that all of them have effective learning capabilities. In contrast, the proposed QGNN has a faster convergence speed and the highest final steady-state reward value, followed by GCN, while RNN performs the worst. This indicates that QGNN can more effectively characterize the collaborative relationship between nodes by introducing the dynamic edge-convolution mechanism, thus achieving better scheduling performance.

**Figure 5:**
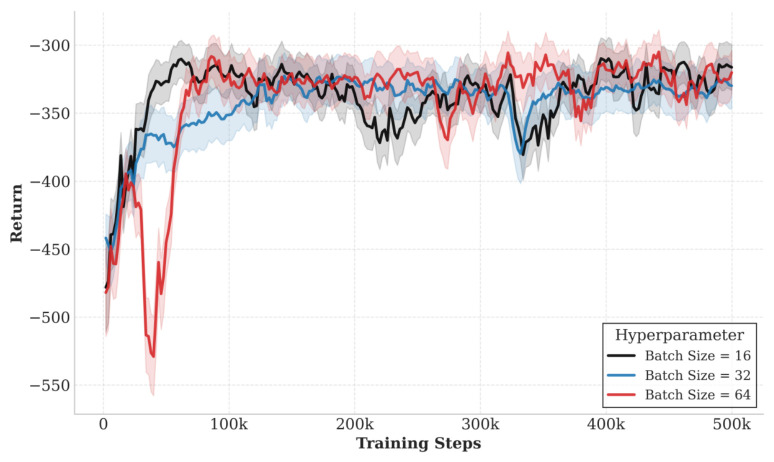
Impact of batch sizes (16, 32, 64) on QGNN training convergence.

Figure 5 shows the impact of different batch sizes (batch size = 16, 32, 64) on the training convergence performance of the QGNN algorithm. It can be seen that the algorithm can achieve stable convergence under all three settings, but there are differences in convergence speed and steady-state performance. Among them, batch size = 32 achieves a better balance between convergence speed and final reward, with the highest final steady-state reward value. In contrast, batch size = 16 converges faster in the early stage but has large fluctuations during training; while batch size = 64 has a significantly slower convergence speed due to the lower frequency of parameter updates. Based on the above results, this paper selects 32 as the default batch size.

**Figure 6:**
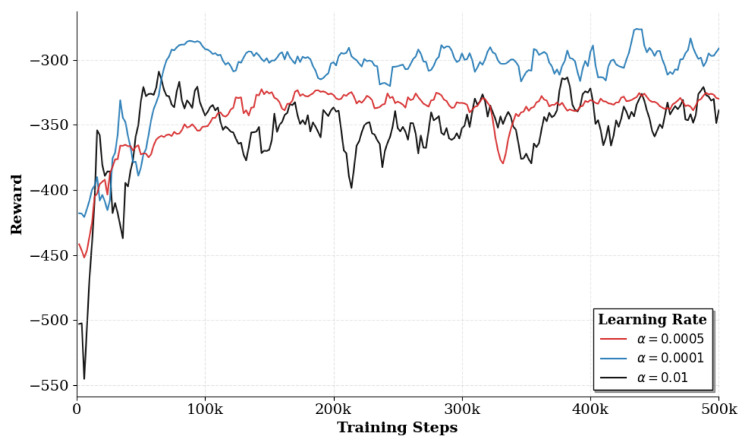
Impact of learning rates (0.0001, 0.0005, 0.01) on QGNN convergence stability.

Figure 6 shows the average reward convergence curves of the QGNN algorithm under different learning rates (Learning Rate = 0.0001, 0.0005, 0.01). It can be seen that the algorithm exhibits a performance improvement trend under all three settings, but there are significant differences in the convergence process and final performance. Among them, when the learning rate is 0.0005, the algorithm achieves the optimal balance between convergence speed and steady-state reward with the best final performance; a smaller learning rate of 0.0001 results in a relatively smooth training process but a slower convergence speed; while a larger learning rate of 0.01 leads to severe fluctuations in the training process, making it difficult to achieve stable convergence. Based on the above results, this paper selects 0.0005 as the default learning rate.

**Figure 7:**
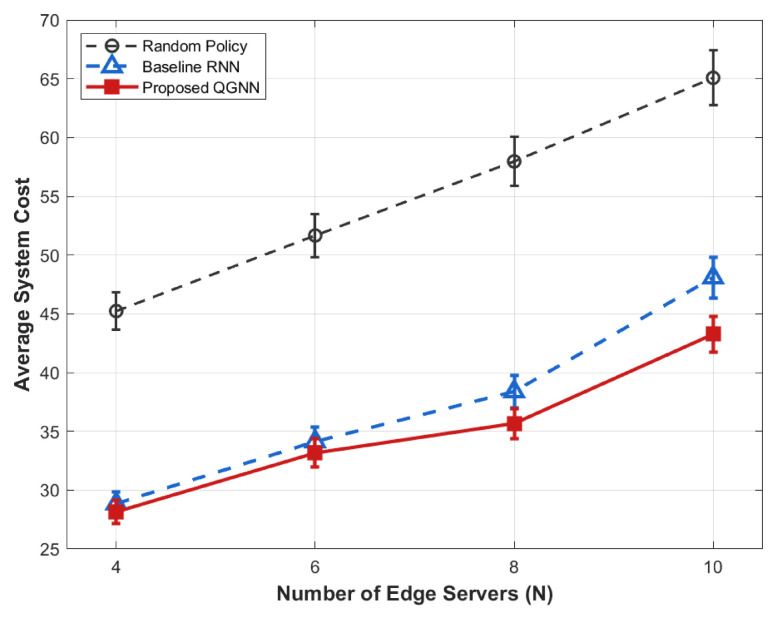
Average system cost of different algorithms under large-scale edge server deployment.

**Figure 8:**
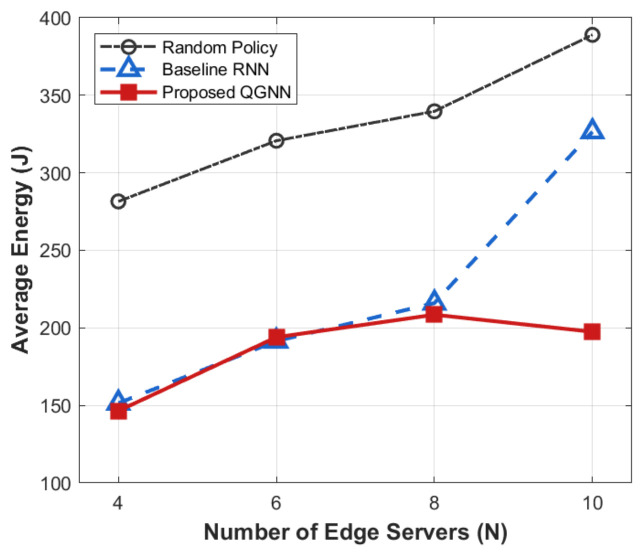
Average energy of different algorithms under varying edge server quantities.

**Figure 9:**
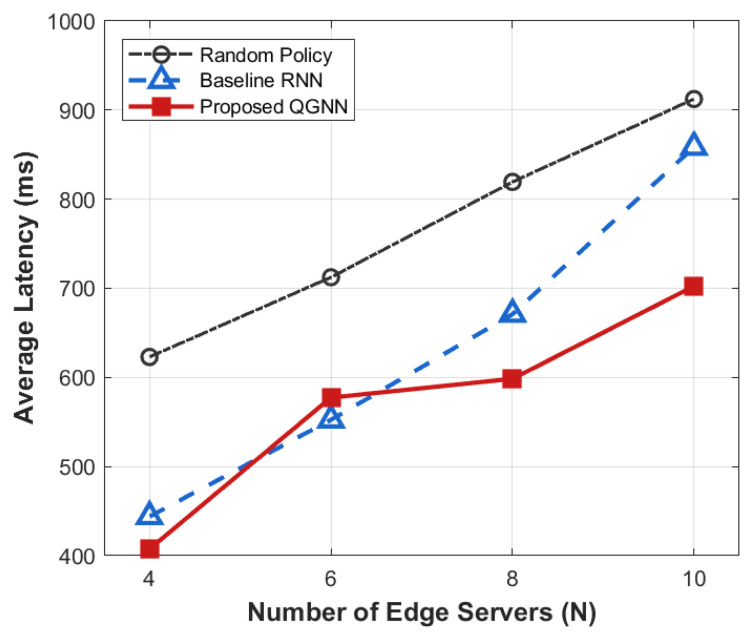
Average latency of different algorithms under diverse edge server scales.

**Figure 10:**
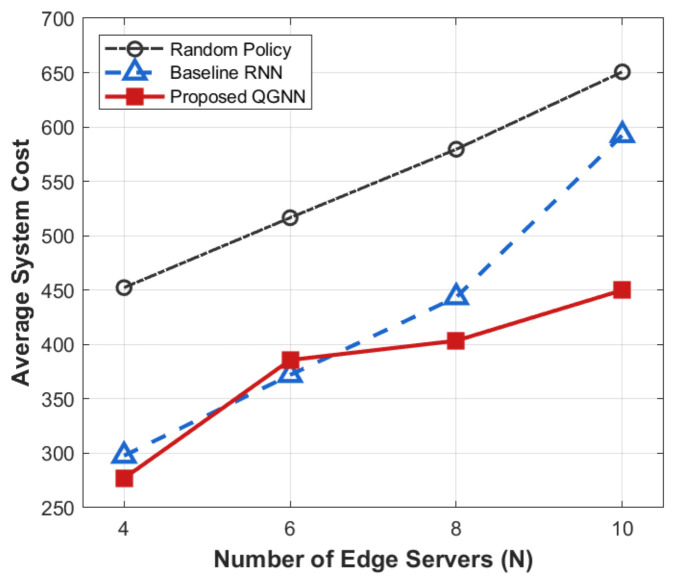
Average system cost of different algorithms under varying edge server scales.

Figure 7, Figure 8, Figure 9 and Figure 10 present the performance comparison results of each algorithm in terms of system overhead, average latency, and average energy consumption under different server node scale conditions, which are used to evaluate the system-level performance of the proposed algorithm under different network scales.

All simulation experiments were independently repeated 10 times, with the results presented as average values. We added 95% confidence intervals to the simulation figures with different horizontal axis variables to demonstrate the statistical stability of the experimental results. For the figures with the same horizontal axis settings, the repeated confidence intervals are omitted to avoid redundancy, and their statistical methods are completely consistent.

From the perspective of the overall trend, as the number of server nodes *N* increases from 4 to 10, the system overhead, average latency, and average energy consumption all show an increasing trend with the expansion of scale. This is mainly because, after the expansion of the network scale, task concurrency, computing resource competition, and communication interaction frequency in the system increase significantly, leading to the continuous accumulation of latency and energy consumption during the scheduling process.

From the perspective of algorithm comparison, the proposed QGNN can maintain low system overhead under different network scales, and its advantages in average latency and average energy consumption are more obvious when the node scale is large. In contrast, RNN and random strategies can maintain acceptable performance in small-scale scenarios, but their system performance decreases significantly as the number of nodes increases.

From the analysis of the reasons for the performance differences, horizontally, the increase in system indicators with the number of nodes is an inevitable scale effect in large-scale industrial IoT scenarios, reflecting the physical cumulative characteristics of system load and resource consumption. Vertically, the performance differences among different algorithms in large-scale scenarios are mainly due to the differences in their scheduling decision mechanisms. RNN and random methods rely on local states for decision-making and lack the ability to effectively perceive the global load distribution, making them prone to uneven resource allocation and queue congestion after network scale expansion. In contrast, the proposed QGNN jointly models the topological relationships and load information between servers through graph neural networks, enabling agents to explicitly consider the collaborative relationships between nodes in the decision-making process, thus achieving more balanced task scheduling and resource allocation in large-scale complex networks and showing better system stability and scalability.

**Figure 11:**
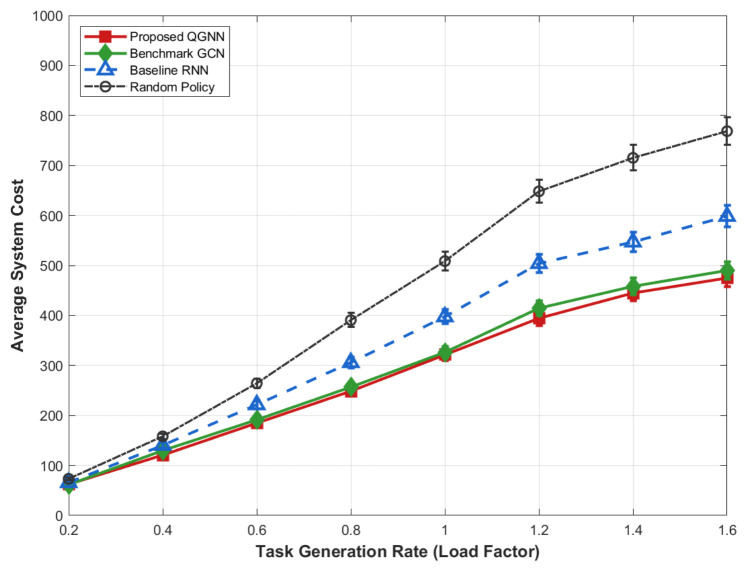
Average system cost of algorithms under varying task load factors (0.2–1.6).

Figure 11 shows the performance of each algorithm in terms of system overhead, average latency, and average energy consumption under different task load factors and is used to evaluate the system robustness and stability of the proposed algorithm in high-load industrial Internet of Things scenarios, serving as the core basis for verifying the basic performance of the algorithm.

**Figure 12:**
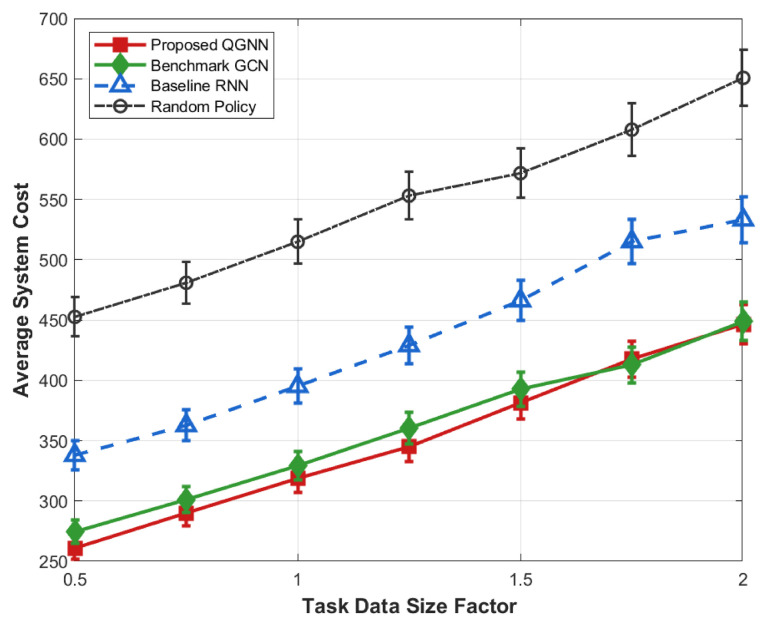
Average system cost of algorithms under different task data size factors.

**Figure 13:**
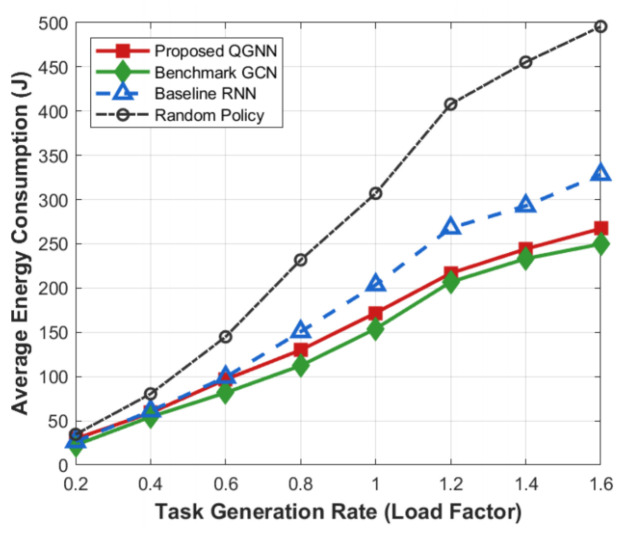
Average energy consumption of algorithms under varying task load factors (0.2–1.6).

**Figure 14:**
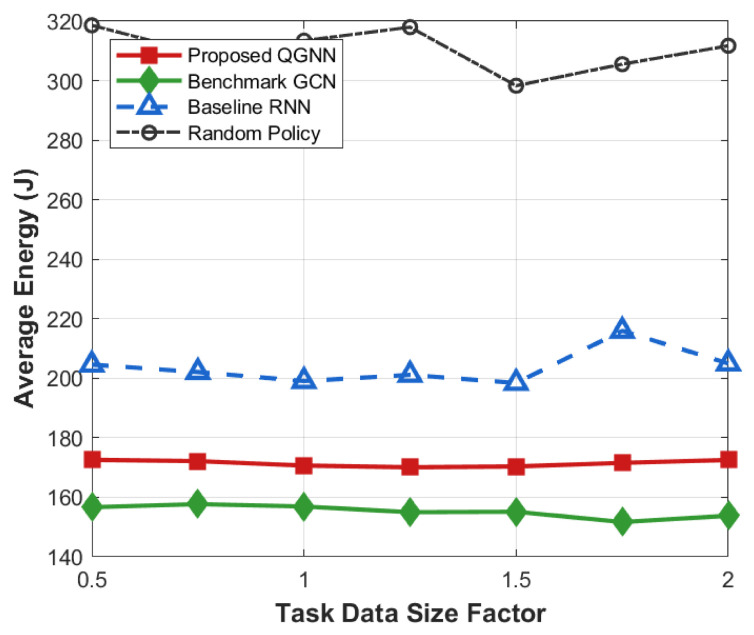
Average energy of algorithms under varying task data size factors.

**Figure 15:**
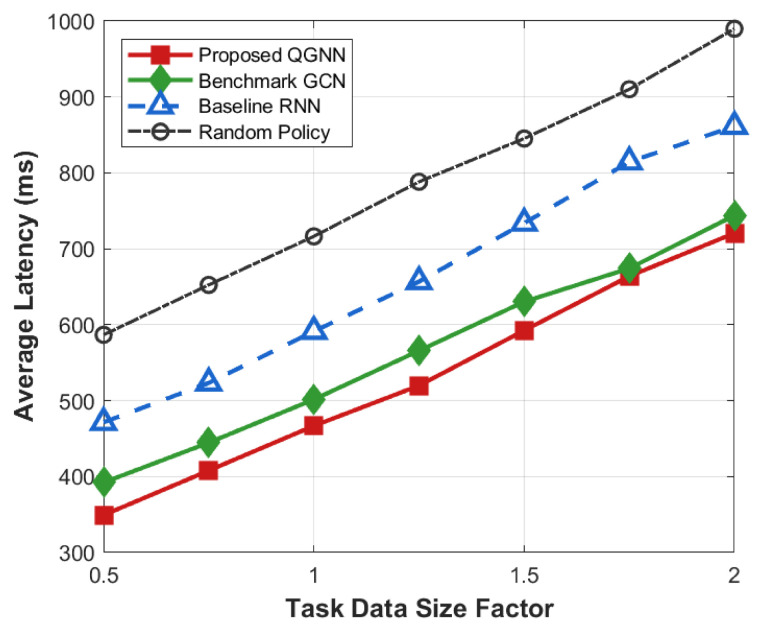
Average latency of algorithms under diverse task data size factors.

Figure 12, Figure 13, Figure 14 and Figure 15 further analyze the performance of each algorithm under variable task loads from the dimensions of system overhead, average latency, and average energy consumption, comprehensively verifying the stability and superiority of the proposed QGNN algorithm in high-fluctuation and high-load industrial Internet of Things scenarios. Meanwhile, the performance gaps between the proposed algorithm and benchmark algorithms are clearly compared, which fully proves the collaborative optimization ability of the algorithm under complex constraints.

From the perspective of the overall trend, as the task load factor increases gradually from 0.2 to 1.6, the average system overhead, average latency, and average energy consumption of all algorithms show an obvious upward trend. This is because after the load level increases, the number of tasks to be processed in the system continues to grow, the computing queues of servers are lengthened, and the task queuing latency accumulates synchronously with computing and communication energy consumption, thus leading to a continuous increase in the system operation cost.

From the perspective of algorithm comparison, the proposed QGNN maintains the lowest or near-lowest system overhead throughout the entire load interval, and its performance degradation rate is significantly slower than that of the comparison algorithms. Especially in the high-load region (Load > 1.0), the system overhead, latency, and energy consumption of RNN and random strategies rise rapidly, while the proposed QGNN can still maintain a relatively stable performance level. Under heavy-load conditions (Load = 1.6), QGNN is significantly superior to GCN, RNN, and random strategies in terms of average latency and energy consumption, indicating that it has stronger scheduling capabilities in high-pressure task scenarios.

From the analysis of the reasons for performance differences: horizontally, the increase in system indicators with the load factor reflects the continuous squeeze on computing resources and communication resources by highly concurrent task flows, which is an inevitable operational characteristic in high-load industrial Internet of Things environments. Vertically, the performance differences among different algorithms under high-load conditions are mainly due to their different capabilities to perceive and make decisions based on the global load state. RNN and random methods mainly rely on local or short-term state information for scheduling, and it is difficult for them to effectively avoid congested nodes when the load is high, which makes them prone to task accumulation and queue amplification effects, leading to rapid deterioration of latency and energy consumption. In contrast, the proposed QGNN jointly models the load distribution and processing capacity among server nodes by introducing a graph-based dynamic feature aggregation mechanism, enabling scheduling decisions to explicitly consider the global resource state. Thus, it realizes adaptive task offloading, effectively suppresses local congestion and nonlinear performance degradation, and exhibits better system robustness and energy efficiency in high-load scenarios.

**Figure 16:**
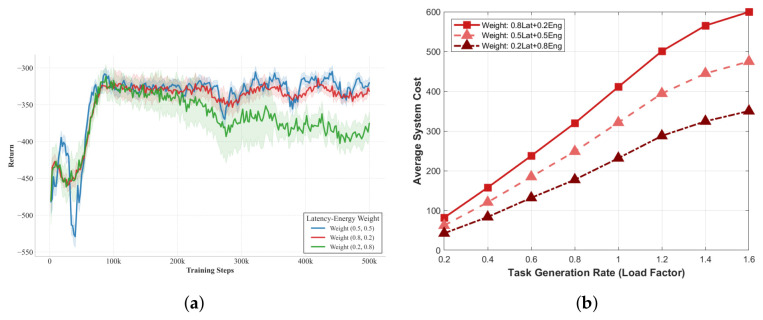
Analysis of latency-energy weight settings and their impact on system performance. (**a**) Rationality analysis of balanced latency-energy weight (0.5:0.5). (**b**) Performance comparison under three latency-energy weight configurations.

To meet the balanced optimization requirements of industrial IIoT scenarios, this paper tentatively sets the weights of latency and energy consumption to 0.5:0.5, giving equal consideration to both latency (closely tied to the QoS of real-time tasks) and energy consumption (related to device battery life and operational costs) to avoid excessive bias toward a single indicator. As shown in Figure 16a, among the comparative results of three weight configurations, the balanced 0.5:0.5 setup achieves a relatively high and stable training return, while the latency-prioritized (0.8:0.2) and energy-prioritized (0.2:0.8) configurations yield lower returns with varying degrees of fluctuation, verifying the impact of different weight settings on latency performance and constraint penalty triggers. Simulations also confirm that the two metrics have comparable magnitudes (10^2^ 10^3^), reducing the risk of dimensional dominance, and that the weights can be flexibly adjusted after normalization for practical scenarios with notable dimensional gaps. As illustrated in Figure 16b, the latency-biased weight (0.8Lat + 0.2Eng) results in the highest system cost by excessively amplifying latency’s impact on the overall overhead, while the energy-biased weight (0.2Lat + 0.8Eng) achieves the numerically lowest cost at the expense of real-time performance that fails to meet the latency QoS requirements of industrial IoT. The balanced weight (0.5Lat + 0.5Eng) strikes an optimal trade-off between the two equally critical objectives of industrial edge computing, aligning with practical industrial demands and ensuring objective algorithm performance evaluation. Thus, this paper adopts 0.5:0.5 as the standard weight for all experiments.

### Conclusions

This paper addresses the decision-making dilemmas and performance degradation in IIoT edge computing multi-server collaborative scheduling. We construct a cloud-edge-end collaborative architecture, model the joint optimization of task offloading, caching update, and resource allocation as a POMDP, and propose QGNN—a graph-guided multi-agent RL algorithm integrating GNNs and QMIX. Simulations show that the proposed method, by capturing node topology and load correlations via GNNs and enabling “centralized training and distributed execution” with QMIX, achieves stable convergence and outperforms GCN and RNN in terms of system overhead, latency, and energy consumption, while exhibiting good robustness and scalability. However, this study relies on idealized industrial topology (excluding extreme dynamic scenarios like frequent device mobility) and lacks business-level indicators (e.g., task completion rate), limiting its direct applicability to complex real-world industrial settings.

This study has limitations: the experiments rely on an idealized topology (excluding extreme dynamic scenarios) and ignore practical high uncertainty and information loss; the GNN may incur extra overhead in large-scale clusters; the optimization objective lacks business-level indicators; and the comparison algorithms exclude emerging distributed methods. Future work will explore multi-indicator dynamic weight optimization, introduce supplementary metrics (e.g., task completion rate and resource utilization), and expand the set of comparison algorithms to provide more persuasive conclusions. Meanwhile, we will also consider dynamic weight calculation combined with different heuristic methods to further improve the optimization effectiveness [33,34,35].

## Figures and Tables

**Table 1:** Nomenclature of Key Symbols.

Symbol	Description	Definition/Notes
K	Set of IIoT devices	{1,2,…,K}, total *K* devices
N	Set of edge server units	{1,2,…,N}, base station + gateway + server
F	Set of task types	{1,2,…,F}, total *F* task categories
$V_{t}^{f}$	Vertex set of system graph	All edge nodes for task *f* at time slot *t*
$E_{t}^{f}$	Edge set of system graph	Interconnections between edge nodes for task *f*
$I_{f}$	Task data size	Data volume of task type *f* (Mbits)
$S_{f}$	CPU cycles for task execution	Computing resources required for task *f* (Gcycles)
$D_{f}$	VNF size for task type *f*	Storage space of the VNF corresponding to task *f*
$N_{k}^{f}\left(t\right)$	Number of type-*f* tasks	Generated by device *k* at time slot *t*
$\mu_{k}^{\left(t\right)}$	Task request state	0: no request; f∈F: type-*f* request
$Q_{n}^{max}$	Maximum computing power	Edge server *n*’s maximum CPU capacity (GHz)
$Q_{k}^{L}$	Local computing capability	IIoT device *k*’s own computing power (GHz)
$Q_{n}^{f}\left(t\right)$	Allocated computing power	For type-*f* tasks on edge server *n*
$Q_{n}^{r}\left(t\right)$	Remaining computing power	Edge server *n*’s unoccupied computing resources
$R_{n,n^{\prime }}^{max}$	Maximum link bandwidth	Between edge node *n* and $n^{\prime }$ (Mbps)
$R_{n,n^{\prime }}^{f}\left(t\right)$	Allocated bandwidth	For type-*f* tasks between node *n* and $n^{\prime }$
$C_{n}^{max}$	VNF deployment capacity	Edge server *n*’s maximum VNF storage (Mbits)
$\alpha_{k}^{\left(t\right)}$	Offloading decision	0: local computing; 1: edge offloading
$b_{f,n}^{\left(t\right)}$	VNF caching state	0: not deployed; 1: deployed on server *n*
$\beta_{f,n}^{\left(t\right)}$	VNF update action	−1: remove; 0: keep; 1: add
$N_{m,n}^{f}\left(t\right)$	Scheduled task number	Type-*f* tasks from node *m* to *n*
T(t)	Total system latency	Sum of transmission, computing, and caching delays (s)
E(t)	Total energy consumption	Sum of device and server energy usage (J)
A(t)	Weighted total cost	γT(t)+(1−γ)E(t)
γ	Cost weight coefficient	Balances latency and energy consumption (0.5)
τ	Time slot length	Task completion deadline (s)
$r_{k}\left(t\right)$	Device uplink rate	IIoT device *k*’s data upload rate (Mbps)
$G_{k}\left(t\right)$	Wireless channel gain	Time-varying gain of device *k*’s channel
$T_{m,n}^{f,tran}\left(t\right)$	Inter-edge transmission delay	For type-*f* tasks from *m* to *n*
$E_{m,n}^{f,tran}\left(t\right)$	Transmission energy consumption	For type-*f* tasks on link (m,n)
$S_{k}^{t}$	Device agent state	$\left\{\mu_{k}^{\left(t\right)},N_{k}^{f}\left(t\right),G_{k}\left(t\right)\right\}$
$S_{n}^{t}$	Server agent state	$\left\{N_{n}\left(t\right),Q_{n}^{r}\left(t\right),b_{n}\left(t\right)\right\}$
$a_{k}^{t}$	Device agent action	Offloading decision $\alpha_{k}\left(t\right)$
$a_{n}^{t}$	Server agent action	$\left\{Q_{n}\left(t\right),N_{n,n^{\prime }}\left(t\right),\beta_{n}\left(t\right)\right\}$
R(t)	Global reward function	Negative cost + constraint violation penalties

**Table 2:** GNN layer structure overview.

Layer	Input Dimensions	Activation Function	Aggregation Method
Input Layer	$x_{n}^{\left(0\right)}=x_{t}$	None	None
Message Generation	$x_{n^{\prime }}^{\left(l-1\right)}$, $e_{n^{\prime },n}$	ReLU	Message Passing
Message Aggregation	Messages from neighbors $m_{n^{\prime },n}^{\left(l\right)}$	None	Sum Aggregation
State Update	$x_{n}^{\left(l\right)}$, $x_{n}^{\left(l-1\right)}$	ReLU	None
Final Layer	$x_{n}^{\left(L\right)}$	None	None

**Table 3:** Simulation environment parameters and reinforcement learning hyperparameters.

Symbol	Description	Value
Industrial Edge Environment Parameters		
*N*	Number of edge servers	4, 6 (default), 8, 10
*M*	Number of devices covered per server	4
$F^{\mathrm{edge}}$	Computing power of edge servers	{1.0,8.0} GHz
$F^{\mathrm{local}}$	Computing power of local devices	{0.2,1.0} GHz
$B^{\mathrm{up}}$	Wireless uplink bandwidth	10 Mbps
$B^{\mathrm{back}}$	Wired backhaul bandwidth	50/500 Mbps
$D_{k}$	Task data size	{1,2,5} Mbits
$C_{k}$	Number of task computing cycles	{0.2,0.5,1.0} Gcycles
$\omega_{t},\omega_{e}$	Weights of the cost function	0.5, 0.5
Reinforcement Learning Hyperparameters		
α	Learning rate	$5\times 10^{-4}$
γ	Discount factor	0.99
$B_{\mathrm{size}}$	Batch size	32
$N_{\mathrm{buffer}}$	Experience replay buffer size	5000
$N_{\mathrm{hidden}}$	GRU/GNN hidden layer dimension	64
σ	Exploration rate ($\sigma_{-greedy}$)	1.0→0.05
$T_{\mathrm{update}}$	Target network update period	200 steps

## Data Availability

The data presented in this study are available upon request from the corresponding author. The data are not publicly available because the research data are confidential.
